# The roadmap for the Allergology specialty and allergy care in Europe and adjacent countries. An EAACI position paper

**DOI:** 10.1186/s13601-019-0245-z

**Published:** 2019-01-24

**Authors:** N. Fyhrquist, T. Werfel, M. B. Bilò, N. Mülleneisen, R. Gerth van Wijk

**Affiliations:** 1 Unit of Systems Toxicology, Institute of Environmental Medicine, Karolinska Institutet, Stockholm, Sweden; 2 Department of Dermatology and Allergy, Hannover Medical University, Hannover, Germany; 3 Allergy Unit, Department of Internal Medicine, University Hospital Ospedali Riuniti, Ancona, Italy; 4 Asthma and Allergy Center, Leverkusen, Germany; 5 Section of Allergology, Department of Internal Medicine, Erasmus Medical Center, Rotterdam, The Netherlands

**Keywords:** Allergology, Specialty, Subspecialty, Allergy, Allergy care, Training

## Abstract

The high prevalence of allergic diseases warrants for sufficient health care provisions available to patients with allergic diseases. Allergy care should be delivered by well-trained specialists. However, the current status of allergy care is not well documented. For this reason a survey among European and a few non-European countries was launched by the National Allergy Society Committee from the European Academy of Allergy and Clinical Immunology and the Union Européenne des Médecins Spécialistes section and board of Allergology. The survey shows that in the vast majority of countries allergy care services are available. However, a substantial heterogeneity is reported regarding recognition of the full specialty, the number of practicing specialists or subspecialists, and training aspects. Growth but also decline of specialty and subspecialties is reported. In addition, the survey gives insight in strengths, weaknesses, opportunities and threats in this field. It appears that the recognition of the full specialty determines strength as well as weakness. Aging of specialists combined with a decline in the number of trainees form a major threat. Opportunities are seen in creating awareness for allergy, focus on attracting young physicians. The conclusion is that harmonization of allergy services across Europe is needed. Investment in young doctors, creating new opportunities and lobbying for the full specialty is required.

**Electronic supplementary material:**

The online version of this article (10.1186/s13601-019-0245-z) contains supplementary material, which is available to authorized users.

## Introduction

The prevalence of allergic diseases is high. In Europe the prevalence of allergic rhinitis is ranging from 17 to 29% [1], whereas asthma varies from 1.3 to 11% according to a European survey among children and adults [2]. In a Finnish cohort of children the rate of physician-diagnosed asthma reached 7.1%, hay fever amounted to 6.2% and the frequency of atopic eczema was 26.5% [3]. Recent data from the Swedish BAMSE cohort show that the prevalence of early life food related symptoms (FRS) and food allergy (FA) amount 12.2% and 6.8% respectively. Amongst children with early life FRS, 35.7% had FRS or FA at 16 years, whereas 74.3% of the children with early life FA had FA at 16 years [4]. A meta-analysis estimates the prevalence of food allergy in Europe at 0.1–6% [5]. Allergy to hymenoptera venom, foods and drugs may lead to severe allergic reactions (anaphylaxis) as documented in the European Anaphylaxis Registry [6, 7].

The high prevalence and concomitant burden of allergic diseases requires sufficient health care provisions to meet the needs of allergy patients. In 2006 an EAACI Task Force published a survey on allergy services in Europe [8]. This report gave an overview of countries having or lacking allergy and clinical immunology services. Moreover, it provided an overview of the availabilities of services covered per country (i.e. allergy, autoimmune disease, immunodeficiency’s etc.) Another source of information on allergy services in Europe comprise a survey among 26 European countries done by the UEMS Allergology Section & Board (S&B) [9]. This survey gives a comprehensive overview on specialties, subspecialties, number of allergologists, training aspects, organization of care, use of immunotherapy, guidelines and Continuing Medical Education (CME).

The WAO White Book on Allergy, update 2013 [10] offers a comprehensive overview of allergic diseases, risk factors, diagnosis and management, prevention, health economics and medical education. Part of this document is a worldwide Member Societies Survey report. Each member society reported data on allergic diseases (prevalence, triggers, socio-economic costs), and allergy care (treatment & training: recognition of the specialty, general practice training and recommendations for improvement).

The European Academy of Allergy and Clinical Immunology (EAACI) has the mission to provide the most efficient platform for scientific communication and education in the field of allergy and immunology ultimately striving to ease the suffering of patients who have these diseases. Promoting good patient care [11] requires insight in the current state of allergy services. Identification of barriers and opportunities are needed to improve health care provisions.

The Union Européenne des Médecins Spécialistes (UEMS) Section and Board (S&B) of Allergology and the EAACI National Allergy Societies Committee (NASC) joined forces to establish and send out a questionnaire to the UEMS delegates and National Society representatives. With this survey we aim to get information on the presence of specialties, subspecialties, number of physicians currently practicing in allergy, and the mobility of specialists across borders in Europe. Moreover, we surveyed the extent of connection between Allergology (training) and clinical immunology, as well as the organization of allergy care and insurance/reimbursement policies. For the first time we also aim to establish a SWOT analysis to identify strengths, weaknesses, opportunities and threats in this area.

## Methods

In 2015 an EAACI Task Force “Allergy services in Europe—an update” to be carried out by the Specialty committee together with the UEMS S&B Allergology was approved. In January 2016 and November 2016 a questionnaire was established by the Specialty committee and UEMS S&B. This questionnaire was sent out to all UEMS delegates. Separately, in 2016 the NASC designed a questionnaire to collect data from National Societies for their own registry. This questionnaire was sent out in the same year. In 2017 it was decided to merge both questionnaires and to send this second survey to UEMS S&B delegates and NAS representatives together. In case of discrepancies between answers from UEMS and NAS representatives from a particular country, respondents were asked to reach consensus. For one country we calculated the mean number of specialists obtained from different respondents. Data collection was done by Nanna Fyhrquist, scientific secretary of the EAACI NASC and Norbert Mülleneisen, secretary of the UEMS S&B. Data cleaning was done by the authors, 3 representatives from the NASC, Nanna Fyhrquist (NF), Thomas Werfel (TW), Maria Beatrice Bilò (MBB) and 2 representatives from UEMS, Roy Gerth van Wijk (RGvW) and Norbert Mülleneisen (NM). A selected number of questions was used to write this executive summary.

By merging the replies from countries in the European community by UEMS representatives with the answers from the NAS the survey goes beyond the borders of the European community. For simplicity “Europe” and “European” in the text refers to all responding countries in and adjacent to Europe.

## Results

### Specialty and subspecialty

**Fig. 1:**
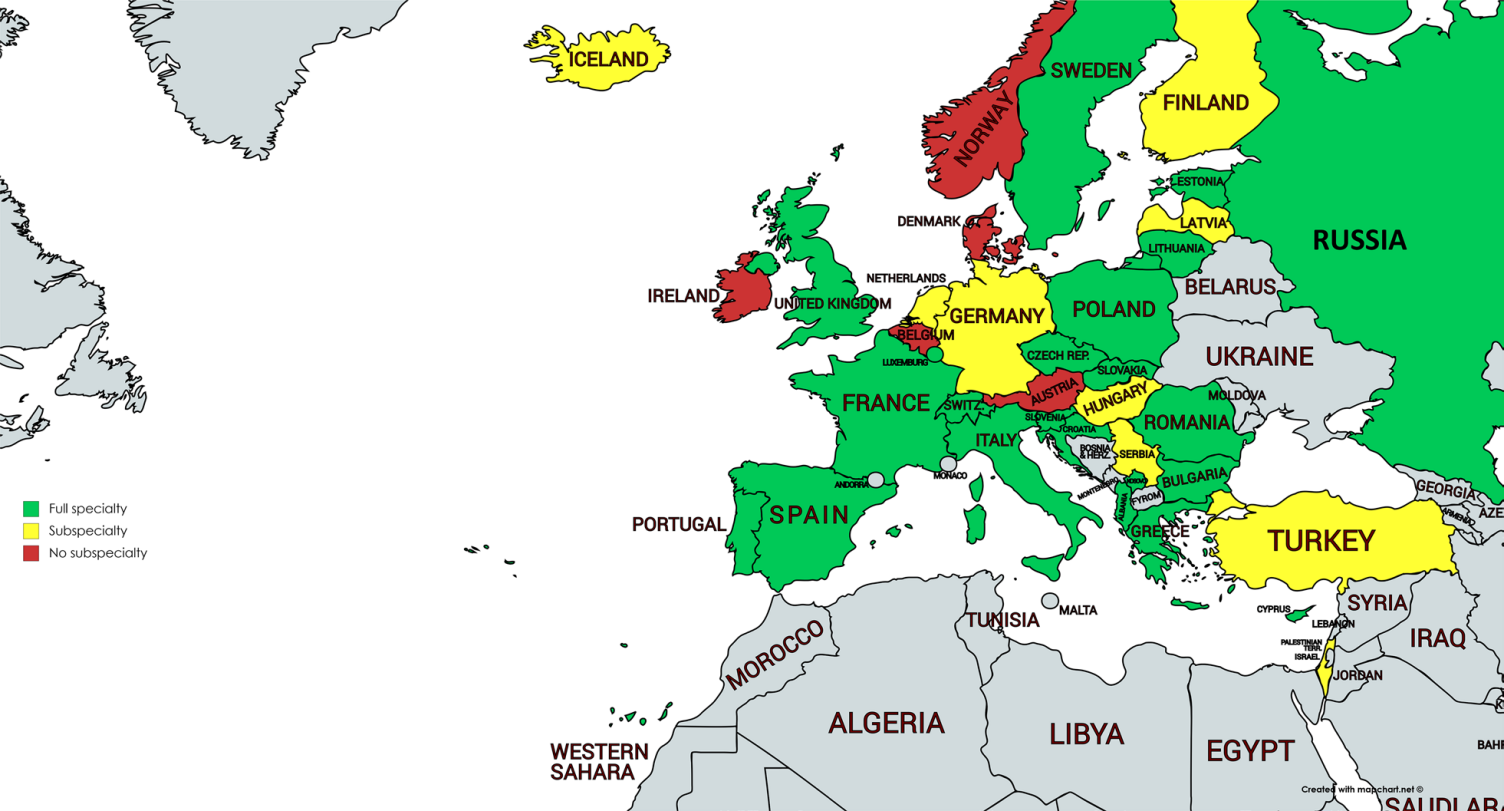
Overview of countries with a specialty (green), subspecialty (yellow) or without a (sub)specialty (red)

Questionnaires were sent to the 51 members of the NASC and the 30 countries linked with the UEMS Allergology S&B. Replies from 36 countries were collected. Figure 1 shows an overview of specialties and subspecialties in Europe. Those countries with both specialties and subspecialties of Allergology are taken together with countries with only a specialty of Allergology. Twenty-three countries reported the recognition of a specialty in their country. Nine countries reported the presence of subspecialties only, five countries do not have a specialty or subspecialty at all (Fig. 1). In Norway a 2 years training for general practitioners, occupational physicians, internists, dermatologists, ENT specialists, pulmonologists and pediatricians is available leading to accreditation in a competence of Allergology. In Denmark there are training possibilities in adult Allergology (2 years) and Pediatric Allergology (3 years), however this training is not recognized as official specialty or subspecialty by the Danish National Medical Association (NMA). Some countries are in a transitional phase. France, Estonia and Slovenia acknowledged the specialty of Allergology recently. Thus, in these countries, the number of already existing subspecialists exceeds the (growing) number of registered allergologists. In contrast, in the Netherlands the specialty lost recognition in 1996, and the declining number of specialists is exceeded by a growth of subspecialists.

To compare the availability of allergologists and subspecialists for patients, apart from the absolute number of allergologists, we also calculated the number of allergologists per 100.000 inhabitants using population data from Worldometers [12] (Table 1). A wide variation can be seen from 6.39 to 0.05 specialists per 100.000 inhabitants in Georgia and the UK respectively (mean 1.81). Also the number of subspecialists varies extensively from 15.9 to 0.15 per 100.000 inhabitants in Finland and the UK (mean 1.84).

**Table 1:** Number of specialists and subspecialists in Allergology

Country	Practising allergologists absolute and per 100,000 inhabitants	
	Full specialty	Subspecialty
Albania	48 (1.64)	
Austria		
Belgium		
Bulgaria	74 (1.04)	
Croatia	29 (0.69)	60 (1.43)
Cyprus	2 (0,17)	
Czech Republic	350 (3.30)	
Denmark		
Estonia	3 (0.23)	15 (1.15)
Finland		110 (1.99)
France	520 (0.80)	1300 (2.00)
Georgia	233 (5.96)	
Germany		4962 (6.04)
Greece	148 (1.33)	
Hungary		500 (5.14)
Iceland		12 (3.58)
Ireland		
Israel		90 (1.07)
Italy	1630 (2.74)	
Kosovo	17 (0.95)	
Latvia		25 (1.28)
Lithuania	60 (2.08)	8 (0.28)
Luxembourg	1 (0.17)	15 (2.57)
Netherlands	4 (0.02)	37 (0.22)
Norway		
Poland	1200 (3.14)	
Portugal	250 (2.42)	
Romania	181 (0.81)	
Russia	2000 (1.39)	
Serbia		31 (0.35)
Slovakia	253 (4,65)	
Slovenia		
Spain	1500 (3.24)	
Sweden	70 (0.71)	103 (1.04)
Switzerland	150 (1.77)	
Turkey		316 (0.39)
UK Ewan	30 (0.05)	100 (0.15)

**Fig. 2:**
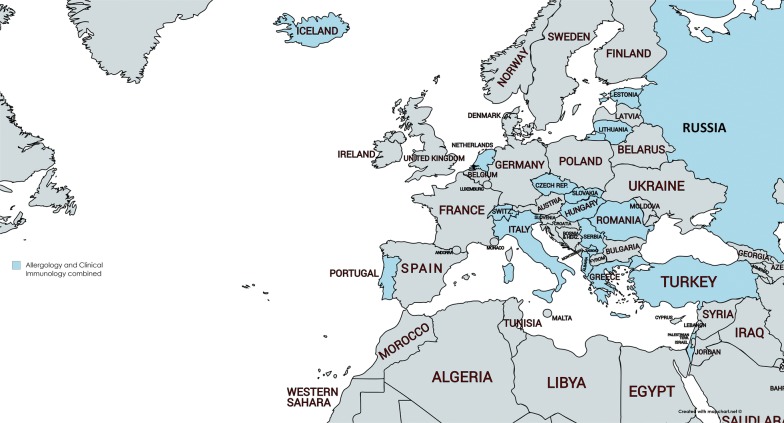
Countries with Allergology and clinical immunology combined in one specialty or subspecialty

In nine countries the presence of both specialties and subspecialties was reported (Table 1). Sixteen countries reported that Allergology and clinical immunology were combined in a specialty or subspecialty (Fig. 2).

The number of yearly registered specialists is associated with the total number of specialists per country. The majority of countries report a yearly registration rate between 1 and 10 new specialists (Additional file 1: Table S1). The top 3 countries are Italy (40–42), Spain (40–55) and Poland (30) Germany is the country with the largest yearly registration of new subspecialists (140), but this number is declining. Respondents also reported whether the specialty or subspecialty is growing, stable or declining (Additional file 2: Figure S1). Most countries reported growth or a stable situation.

**Fig. 3:**
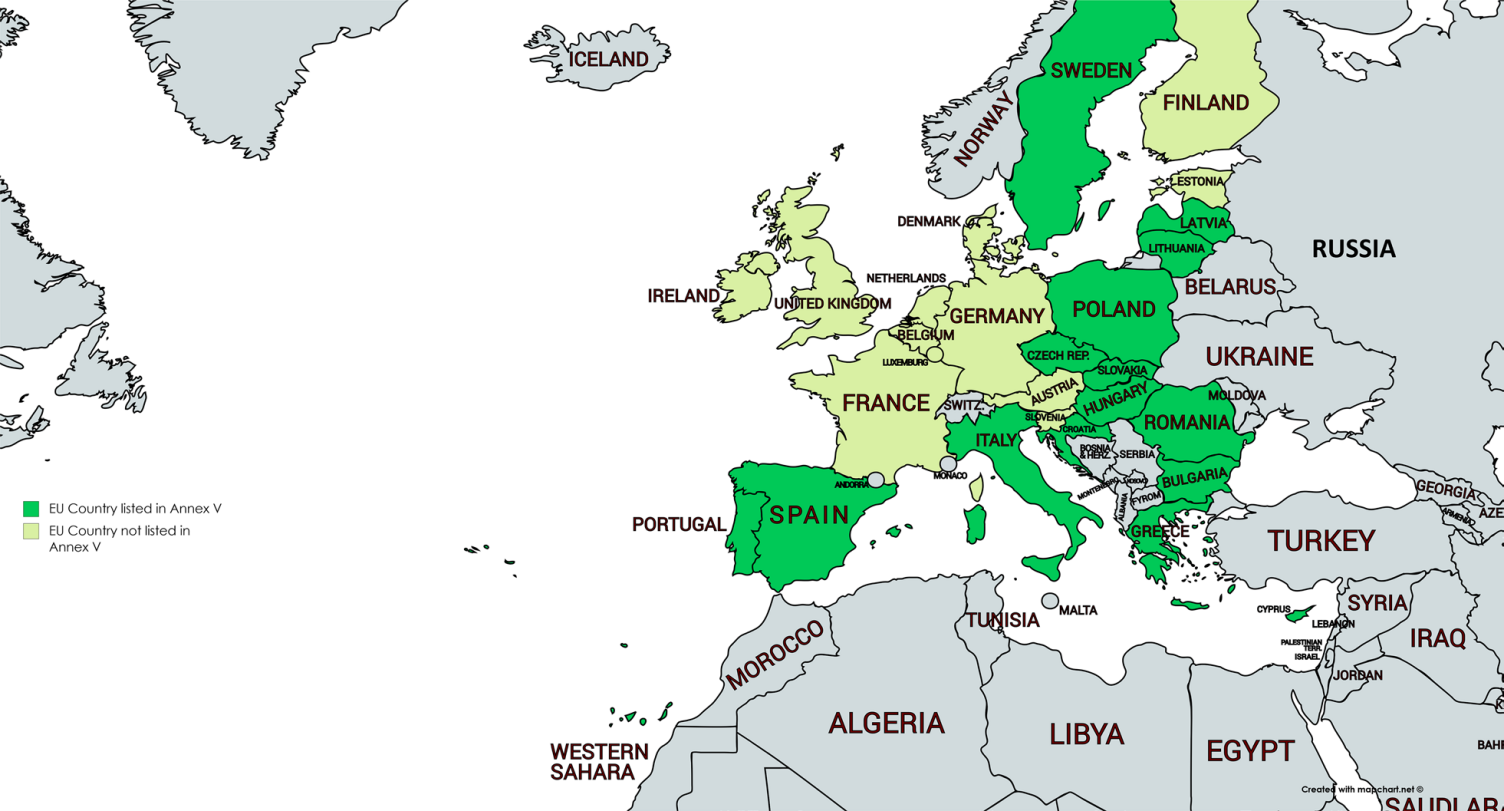
EU member states with the specialty of Allergology listed in Annex V

It is important that European specialists can freely move between countries. Mutual recognition of the specialty by the different countries makes that possible. A condition is that a specialist medical qualification is listed in Annex V of the Directive on Recognition of Professional Qualifications. This list has been amended in 2016 [13]. Not all countries with a specialty of Allergology have the specialist medical qualification (comprising title and minimum duration of training) listed in Annex V (see Fig. 3). Free movement is possible in 15 of 26 EU member states.

### Training in the specialty

**Fig. 4:**
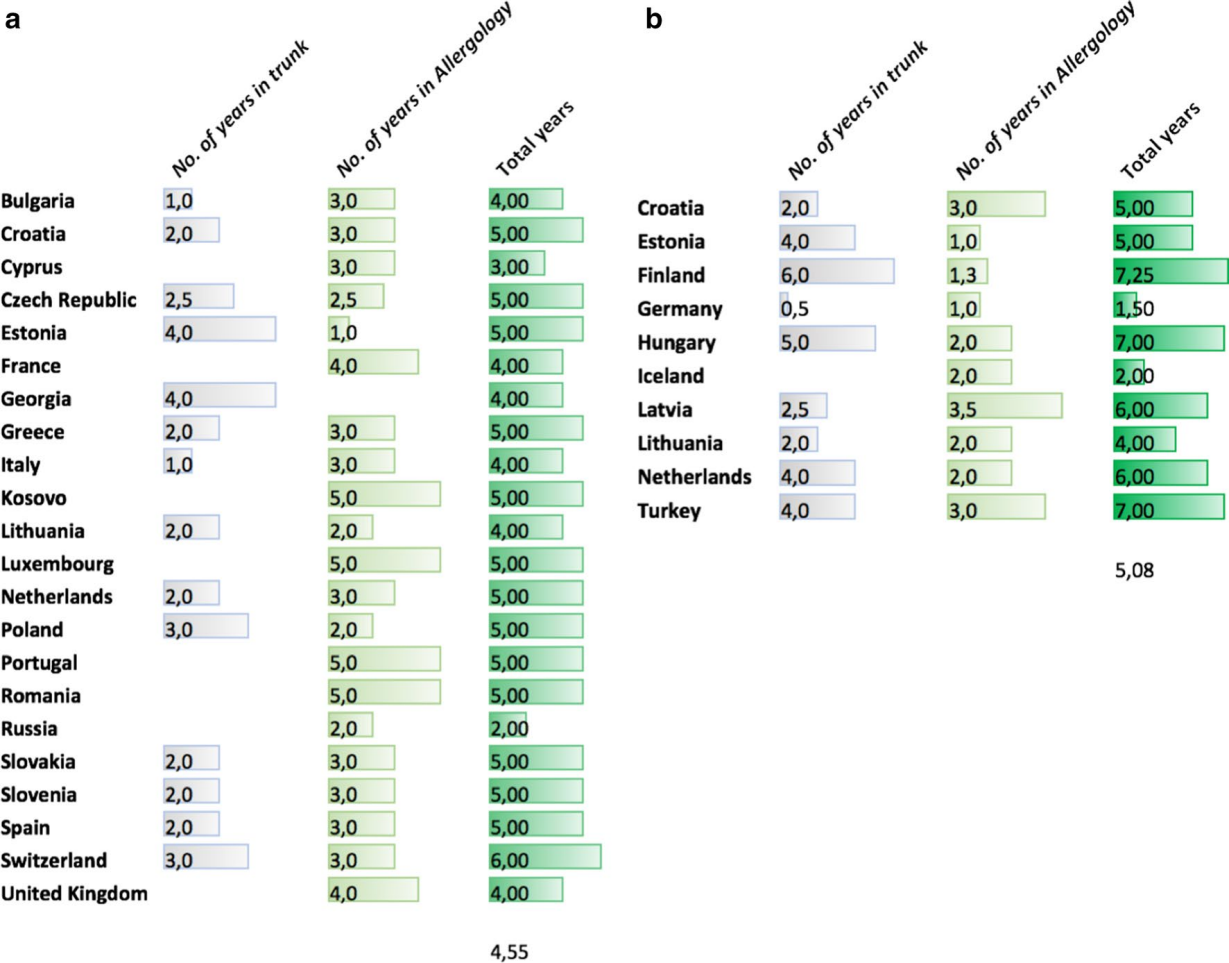
Years of specializations in countries with **a** full specialty and **b** subspecialty in Allergology

Figure 4 shows the training schemes for specialists and subspecialists. The total number of years varies between 1.5 and 7 years. The mean duration of the full specialty and subspecialty amounts 4.52 and 5.08 years respectively. Many delegates report a common trunk ranging from 0.5 to 5.0 years whereas the number of years in Allergology range from 1.0 to 5.0. Some countries such as Portugal with a duration of 5 years include a period of internal medicine or pediatrics in their curriculum. However, these periods are not labeled as common trunk but seen as part of the specific Allergology training.

### SWOT analysis

**Fig. 5:**
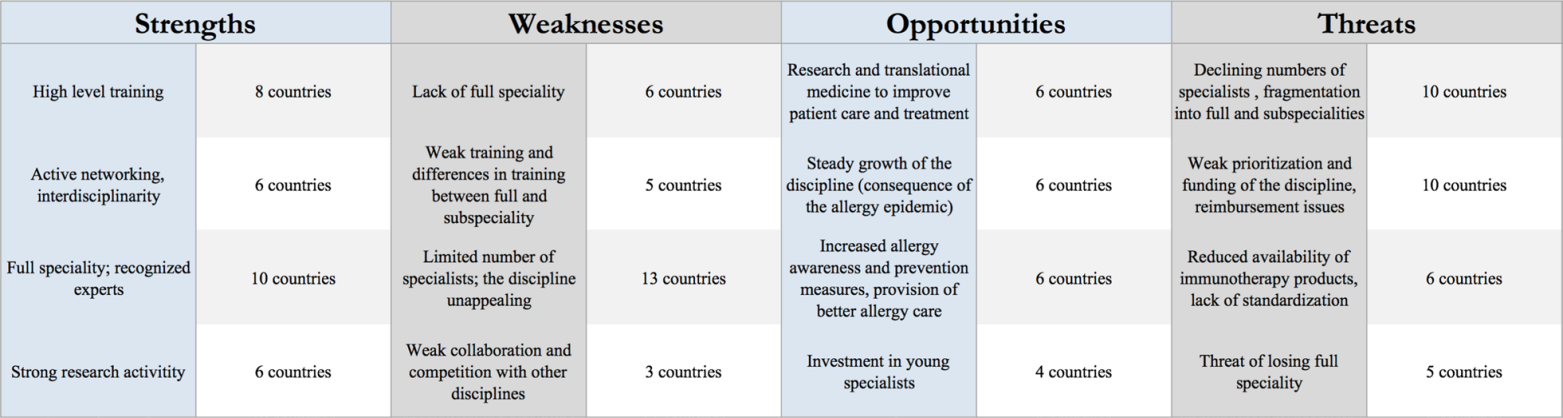
SWOT analysis overview

The SWOT analysis reflects the situation of Allergology at a local level. The presence of a full specialty can be considered as a strength in one country, whereas the lack of such a discipline will be seen as a weakness. In some countries, the loss of a full specialty was listed under threats. Other threats included lack of attractiveness of the discipline among young doctors, as well as its weak prioritization by local authorities. Moreover, limited funding opportunities and insufficient reimbursement policies were considerable challenges, and in some countries the reduced availability of immunotherapy products and lack of standardization in this context, were concerns. Notwithstanding, the continuing growth of the discipline, increased allergy awareness and improved prevention measures, as well as ongoing efforts to improve allergy care, were regarded as opportunities. Finally, research and translational medicine to improve patient care and treatment, as well as the investment in young specialists, were considered as opportunities. Local diversity in policy and circumstances may contribute to the diversity in the answers to the questionnaires. Figure 5 displays the most important topics being put forward by representatives of 3 or more countries.

## Discussion

The survey dispatched to UEMS and EAACI NASC delegates aimed to collect up-to-date information on how Allergology in Europe is organized. The ultimate goal is to get better insight in the level of allergy care, the accessibility for patients to specialists and subspecialists, the barriers and opportunities for the specialty and subspecialties. Such information is needed for the communication with policy makers at a national and international level. Moreover, identifying the unmet needs is required to consider the next steps in order to strengthen the field of Allergology.

An important outcome of the survey is that most countries recognize the full specialty, a minority has one or more subspecialties, whereas a few countries only do not recognize either the specialty or subspecialties. Moreover, many countries report growth or stability in terms of number of (sub)specialists. However, the number of (sub)specialists per 100.000 varies substantially underlining the unequal distribution in allergy care across Europe. In addition, in spite of the high burden of allergic diseases the number is much lower compared to other adjacent specialties. Eurostat reports health care data per specialty and country in Europe. From those tables the mean number of specialists/100.000 can be calculated. It is not surprising that the estimates for dermatologists, otolaryngologists and pulmonologists are much higher (5.84, 6.31 and 4.80 respectively) [14] than the mean number of allergologists and subspecialists in Allergology (1.81 and 1.84). Thus overall, one allergologist or subspecialist is accessible for about 53,000–54,000 persons. For some countries these estimates can be completely theoretical, since many young specialists do not find work as allergologists at the end of their study course. For instance a survey carried out among Italian young specialists showed that more than 50% of young people who registered in the last 5 years does not deal with allergic diseases, but works in other areas such as Emergency Room, Internal Medicine, Transfusion Medicine (personal communication). Furthermore, the decline of Allergology in several countries is a major concern. Particularly, in Germany with already a low training duration, there are plans to further limit the training thereby marginalizing the position of the subspecialty and endangering the care to allergic patients. In some other countries there is also the threat of ending the recognition of the specialty.

In several countries the disciplines of Allergology and Clinical Immunology are combined. This fits in the policy of EAACI to embrace both domains. Also, the EAACI-UEMS exam pays attention to both areas. The survey however does not give insight in the day-to-day practice of specialists trained in both fields. It cannot be excluded that specialists after their registration will only have practice predominantly in one domain.

Free movement of workers is a fundamental freedom enjoyed by EU citizens. However, for allergologists free movement is determined by the presence and the inclusion of the specialty in Annex V. This means that a Spanish allergologist can work as a specialist in Italy or Poland, but not in Germany (subspecialty only) or France and the UK (specialty but not listed in Annex V). By expanding the number of countries with a full specialty and by convincing countries to enlist Allergology on Annex V barriers between countries may be removed. On the other hand, subspecialists can move easily. For instance, a dermatologist with Allergology as subspecialty can move to all countries in Europe as Dermatology is recognized across Europe. It is however possible that the registration in the subspecialty in the new country is not recognized.

This survey also gives global insight in the training schemes for allergologists and subspecialists. According to the requirements for the specialty of Allergology and Clinical Immunology (not an accepted title in annex V) as approved in 1994 and amended in 1997–2003 the training in the specialty should comprise minimally 2 years in a common trunk and minimally 3 years in the specialty including several months of Immunology, Dermatology, Pulmonology and Otolaryngology [15, 16]. From the survey it is clear that many training schemes across Europe deviate from these requirements, and one of many concerns is the unequal and ill-defined training of specialists and subspecialists. The requirements from 2003 need to be updated and modernized according to the current UEMS standards for ETRs (European Training Requirements). In addition, attempts should be made to harmonize national training requirements using the ETR as benchmark. Harmonization was also the main goal of establishing objectives of training and a specialty training core curriculum [15, 16]. On the other hand, one should bear in mind that the states remain free to organize the specialties at a national level.

Apart from the training requirements for pediatric allergologists aiming at a tertiary care level, there are no generally accepted training requirements for subspecialties in Allergology. A recent EAACI position paper has been published addressing this topic [17]. The EAACI Specialty committee considered the lower limit of training at 18 months. From the survey it can be seen that in most countries the duration of training in Allergology exceeds 18 months.

For the first time a SWOT (strengths, weaknesses, opportunities and threats) analysis across Europe has been carried out. In general, SWOT analyses are intended to help with developing a strategic plan for projects or business. Given the heterogeneity in allergy services it is not surprising that also the obtained SWOT analyses—based on the national position and environment of Allergology—substantially differ from country to country. However, common denominators can be extracted from the answers. Moreover, the SWOT analysis reveals opinions on future perspectives that are not captured by the rest of the survey. The recognition of a full specialty is important. The presence of a full specialty is being seen as a strength, the lack is perceived as a weakness. Multidisciplinarity and the connection with immunology are strong and intrinsic characteristics of Allergology. But different disciplines in this field may also lead to non-cooperation and competition. This is also true when both full specialists and subspecialists are recognized in the same country. Major problems put forward are the limited number of specialists and limited career opportunities. Aging of specialists combined with a decline in the number of trainees form a major threat. Reimbursement and funding issues may further accelerate these trends. Reimbursement issues both for patients and physicians have already been reported in a European survey identifying the barriers for allergen immunotherapy in primary care [18]. Moreover, the availability of products for immunotherapy is a concern; due to the scarcity of approved products and the lack of standardization, there are numerous challenges to the allergologist. The question arises what can be done. The respondents see opportunities in spreading out the full specialty, creating awareness among general practitioners and political lobbying. Furthermore, investments in young specialists and creating research opportunities may attract more young physicians. This asks however for intensive lobbying at both a European and national level.

The survey has, however, a few limitations. Ideally, the data collected should be based on accurate registries. However, it could be seen from discrepancies in answers when both EAACI NASC and UEMS representatives replied from one country, that such registries are missing. Discrepancies had to be solved by approaching the respondents again. All in all the numeric data from this survey are sometimes precise and sometimes merely estimations. Furthermore, although the respondents are official representatives of UEMS and EAACI NASC, we do not know to what extent the respondents obtained feedback from their own organizations. For instance, it is possible that the outcome of the SWOT analysis represent the personal view of the respondents.

## Conclusion and unmet needs

This survey clearly shows that in the majority of European and adjacent countries the full specialty of Allergology has been recognized with recent acknowledgements in Estonia, France and Slovenia. However, it is also clear that the spectrum of allergy services is very heterogeneous across Europe. Allergy care varies in the type of caregivers, number of specialists, and training of specialists. More harmonization should be achieved in training of allergologists and subspecialists. When a country has not a full specialty, this is perceived as a weakness, whereas the presence of the specialty is seen as a strength. Investment in young doctors, creating new opportunities and lobbying for the full specialty is needed. In addition, free movement of allergologists should be better facilitated.

## Additional files

**Additional file 1.** Growth, stability and decline of the specialty/subspecialty in Europe.

**Additional file 2.** Number of newly registered specialists or subspecialists per year.
