# Supplementary material for: The roadmap for the Allergology specialty and allergy care in Europe and adjacent countries. An EAACI position paper
Source: Clin Transl Allergy. 2019 Jan 24;9:3. doi: 10.1186/s13601-019-0245-z (PMC6345018; doi:10.1186/s13601-019-0245-z)
Supplement: Supplementary file 1 — Additional file 1. Growth, stability and decline of the specialty/subspecialty in Europe. [file 13601_2019_245_MOESM1_ESM.docx]

Table S1

| Country | number of yearly registrations in the specialty or subspecialty |
| --- | --- |
| Albania | Every 1-2 years 3-5 |
| Bulgaria | ≈ 4 |
| Croatia | 1-2 |
| Cyprus | 0 |
| Czech Republic | 6-10 |
| Denmark | 3 |
| Estonia | 1 |
| Finland | 26 |
| France | No data |
| Germany | ≈140 |
| Greece | 5-7 |
| Hungary | ≈ 5 |
| Iceland | 0-1 |
| Ireland | 0.5 |
| Israel | 3-4 |
| Italy | 40-42 |
| Kosovo | 2-3 |
| Latvia | 0.5 |
| Lithuania | 2-6 |
| Luxembourg | <1 |
| Netherlands | 2-3 |
| Poland | ≈30 |
| Portugal | ≈ 10 |
| Romania | 10-16 |
| Russia | 3-10 |
| Serbia | 1-2 |
| Slovakia | 1-5 |
| Spain | 40-55 |
| Sweden | 3 |
| Switzerland | 6-12 |
| Turkey | 15 |
| UK Ewan | 1 |
