# Supplementary figures and images for: The roadmap for the Allergology specialty and allergy care in Europe and adjacent countries. An EAACI position paper
Source: Clin Transl Allergy. 2019 Jan 24;9:3. doi: 10.1186/s13601-019-0245-z (PMC6345018; doi:10.1186/s13601-019-0245-z)

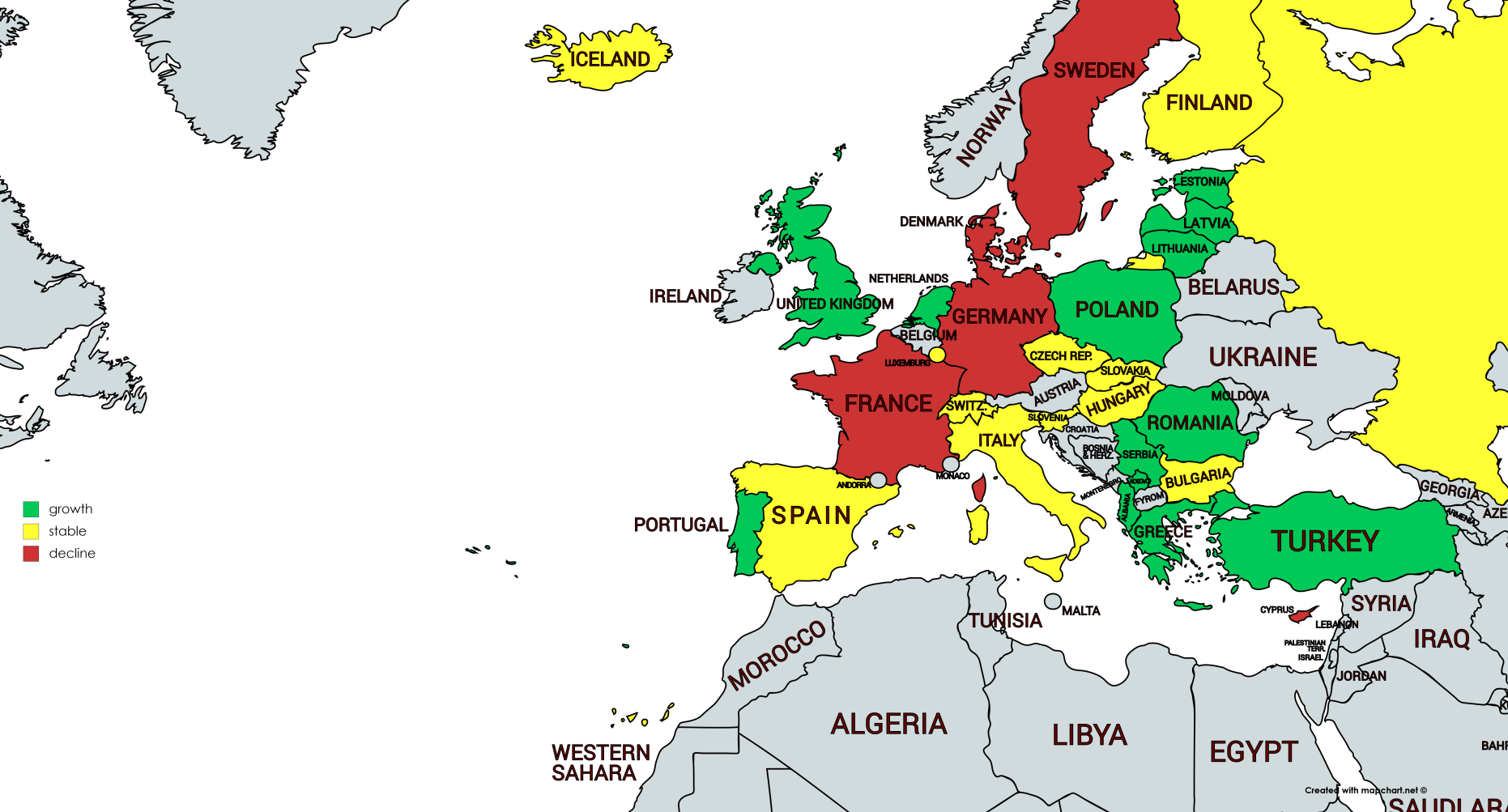


Figure S1

Supplement: Supplementary file 2 — Additional file 2. Number of newly registered specialists or subspecialists per year. [file 13601_2019_245_MOESM2_ESM.docx]
